# High *RPS27A* Expression Predicts Poor Prognosis in Patients With HPV Type 16 Cervical Cancer

**DOI:** 10.3389/fonc.2021.752974

**Published:** 2021-11-02

**Authors:** Qiming Wang, Yan Cai, Xuewen Fu, Liang Chen

**Affiliations:** ^1^ Department of Gynecology, Ningbo Women & Children’s Hospital, Ningbo, China; ^2^ School of Medicine, Ningbo University, Ningbo, China

**Keywords:** RPS27A, HPV type 16 cervical cancer, HPV16, cervical cancer, squamous cell carcinoma

## Abstract

In recent years, the incidence and the mortality rate of cervical cancer have been gradually increasing, becoming one of the major causes of cancer-related death in women. In particular, patients with advanced and recurrent cervical cancers present a very poor prognosis. In addition, the vast majority of cervical cancer cases are caused by human papillomavirus (HPV) infection, of which HPV16 infection is the main cause and squamous cell carcinoma is the main presenting type. In this study, we performed screening of differentially expressed genes (DEGs) based on The Cancer Genome Atlas (TCGA) database and GSE6791, constructed a protein–protein interaction (PPI) network to screen 34 hub genes, filtered to the remaining 10 genes using the CytoHubba plug-in, and used survival analysis to determine that *RPS27A* was most associated with the prognosis of cervical cancer patients and has prognostic and predictive value for cervical cancer. The most significant biological functions and pathways of *RPS27A* enrichment were subsequently investigated with gene set enrichment analysis (GSEA), and integration of TCGA and GTEx database analyses revealed that *RPS27A* was significantly expressed in most cancer types. In this study, our analysis revealed that *RPS27A* can be used as a prognostic biomarker for HPV16 cervical cancer and has biological significance for the growth of cervical cancer cells.

## Introduction

Cervical cancer (cervical squamous cell carcinoma and endocervical adenocarcinoma, CESC) is the fourth most common cancer in women, after breast, colorectal, and lung cancers (1). In the last 2 years, the incidence and the mortality of cervical cancer have been increasing globally, with more than 600,000 new cases and nearly 350,000 deaths in 2020 (2, 3). The age of onset of cervical cancer is “bimodal” and is concentrated in women in their 30s and 40s (4), and about 85% of cervical cancer deaths occur in less developed and developing countries due to medical conditions (5). Patients with early-stage (IB–IIA) cervical cancer overwhelmingly show a trend of good prognosis after receiving appropriate treatment (6), but the prognosis of patients with advanced and recurrent cervical cancer remains poor (7). In addition, cervical cancer is metastatic, with the most frequent site being the bone, and the median survival time after diagnosis is only 7–12 months (8).

Of the 22 million new cancer cases caused by an infection in 2018, up to 690,000 were affected by human papillomavirus (HPV) (9). HPV is a double-stranded DNA virus, and most types of HPV infections are cleared by autoimmunity. However, a few types of HPV viruses can transform infected cells into malignant tumor cells (10). HPV infection is transmitted through sexual contact (early-age sexual intercourse and multiple sexual partners are both high-risk factors for HPV infection), and persistent HPV infection is the most important factor in the development of cervical cancer (11). HPV testing is the primary modality for cervical cancer screening and can significantly reduce the risk of death from cervical cancer (12). In addition, broad-spectrum HPV vaccination is an effective way to prevent the development of cervical cancer (13, 14). To date, three HPV vaccines have been licensed for use: the bivalent HPV virus-like particle vaccine (2vHPV), the quadrivalent HPV virus-like particle vaccine (4vHPV), and the nonavalent HPV virus-like particle vaccine (9vHPV), which can prevent 70% of cervical cancers worldwide (15).

More than 40 HPV virus types colonize the genital tract, 15 of which are associated with cervical cancer, and HPV16 is one of the most virulent genotypes (16). High-risk HPV16 is associated with genital and oropharyngeal cancers (17) and approximately 50% of cases of squamous cell carcinoma (SCC), the most frequent type of cervical cancer (18). Women who have been persistently infected with HPV16 for 2 years have a high probability of developing precancerous lesions within the next 5 years (19), and persistent HPV16 infection is the most important factor leading to the recurrence of high-grade cervical intraepithelial neoplasia (CIN) after treatment in patients with HPV infection (20).

In this study, based on the analysis of The Cancer Genome Atlas (TCGA) database and Gene Expression Omnibus (GEO) database, we aimed to screen the pivotal genes through the screening of differentially expressed genes (DEGs) and the construction of a protein–protein interaction (PPI) network, identify the key gene by analyzing the relationship between the high and low expressions of the pivotal genes and the survival of cervical cancer patients, use this key gene to predict the survival of cervical cancer patients, and analyze the main functional pathways of the key gene using gene set enrichment analysis (GSEA) to determine the prognostic biomarkers for cervical cancer caused by HPV16 infection.

## Information and Methods

### Data Sources

All clinical information and gene expression-related matrix data related to cervical cancer were obtained from TCGA database, GEO database (GSE6791) (https://www.ncbi.nlm.nih.gov/geo/), and the Genotype–Tissue Expression (GTEx) database. TCGA included 12 HPV16-positive samples and 294 HPV16-negative samples, GSE6791 included eight HPV16-positive samples and three HPV16-negative samples, and para cancer tissue data were obtained from GTEx.

### DEG Screening and Functional Pathway Enrichment Analysis

DEGs were screened for HPV16-related genes using the R limma package, and volcano plots were plotted by the R package ggplot2 with log2FC = 0.3785 and *p* < 0.05 as the screening conditions. Gene ontology (GO) and Kyoto Encyclopedia of Genes and Genomes (KEGG) functional pathway enrichment analyses were performed on the screened DEGs using the R package clusterProfiler to explore the biological characteristics of DEGs. Venn diagrams were drawn to identify overlaps between the DEGs in TCGA and GSE6791.

### Selection and Identification of Hub Genes

DEGs were imported into the STRING database (https://string-db.org/) to construct a PPI network, visualized by Cytoscape (version 3.8.2), and pivotal genes were screened by degree sorting using the CytoHubba plugin. The performance of each pivotal gene was observed in TCGA and GSE6791 databases, and the expression levels of the pivotal genes in cervical cancer, cervical SCC, and cervical adenocarcinoma tissues were compared with those in normal tissues. Kaplan–Meier curves were plotted to observe the overall survival (OS) of high and low expressions of pivotal genes, and the genes most associated with the prognosis of cervical cancer patients were selected as key genes.

### Validation of Pivotal Genes

The expression levels of key genes in the different clinical stages were analyzed using the R package ggplot2, and the relationship between the key genes and the different clinical stages, including prognosis, of cervical cancer patients was analyzed by the R package survival. Subsequently, the subject operating curve (receiver operating characteristic, ROC) was plotted to assess the diagnostic value of the expression levels of the key genes for HPV16 positivity. *P* < 0.05 was considered statistically significant.

### Single Gene Set Enrichment Analysis

GSEA is a method for analyzing gene expression data to assess pathway enrichment in transcriptional data (21). The median gene expression was used as a grouping condition, and the biological functions associated with the hub genes in HPV16 were analyzed using the R package clusterProfiler by matching mutual species with the functions in the R package msigdbr. The screening conditions were *p* < 0.05 and false discovery rate (FDR) < 0.2. The presentation was visualized using gseaplot2, a function in the R package clusterProfiler.

### Pan-Cancer Analysis

The data of 33 cancers and normal tissues were obtained from TCGA database and GTEx database to analyze the expression difference between the key genes in cancer and para cancer and observe the association of the gene with other cancers.

## Results

### Screening of DEGs

**Figure 1:**
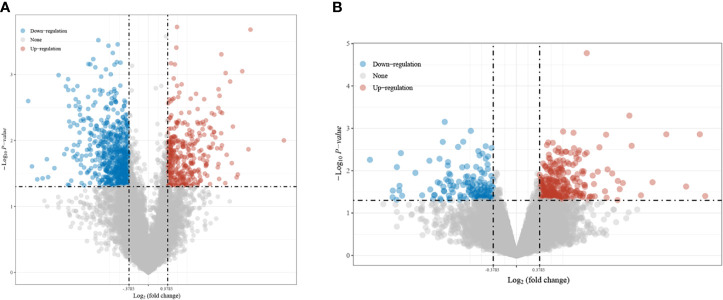
Screening results of the differentially expressed genes (DEGs). **(A)** intersecting genes in TCGA and GSE6791. **(B)** Screened from the differential genes in The Cancer Genome Atlas (TCGA).

In this study, 1,069 DEGs were screened from TCGA, among which 362 genes were upregulated and 707 genes were downregulated (**Figure 1A**). Four hundred and forty-six DEGs were screened from GSE6791, of which 302 genes were upregulated and 144 genes were downregulated (**Figure 1B**).

### GO/KEGG Enrichment Analysis of DEGs

**Figure 2:**
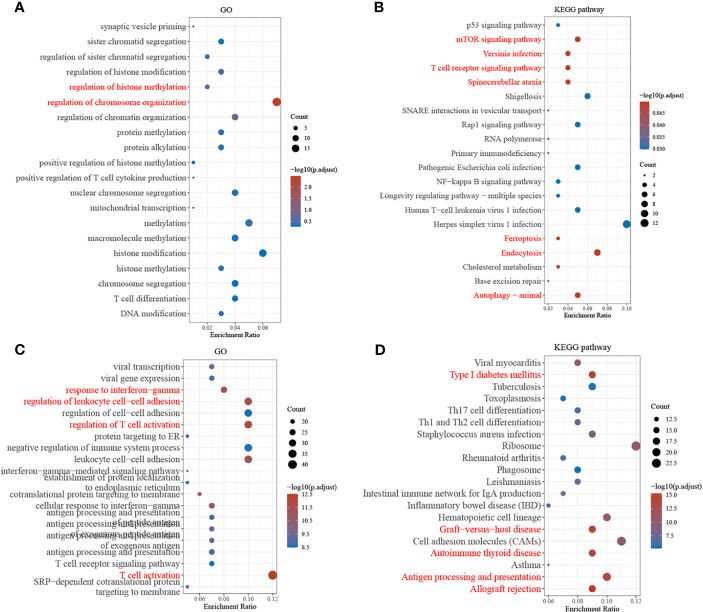
Gene ontology (GO) and Kyoto Encyclopedia of Genes and Genomes (KEGG) analyses of the differentially expressed genes (DEGs). **(A, B)** Screening of GO terms and KEGG pathway for DEGs from GSE6791. **(C, D)** Screening of GO terms and KEGG pathway for the differential genes from The Cancer Genome Atlas (TCGA).

The top 20 GO terms and KEGG pathways with the most enriched upregulated genes in DEGs were listed by *p*-value, as shown in **Figure 2**. In the GSE6791 database, DEGs were mainly enriched in GO functions such as regulation of chromosome organization, histone, and methylation regulation and in KEGG pathways such as the ribosome, tumor necrosis factor signaling pathway, and iron death. In TCGA database, the DEGs were mainly enriched in GO functions such as T-cell activation and regulation, response to interferon−gamma, and regulation of leukocyte cell–cell adhesion and in KEGG pathways such as transplant rejection, antigen outgrowth, and presentation.

### Screening of Pivotal Genes

**Figure 3:**
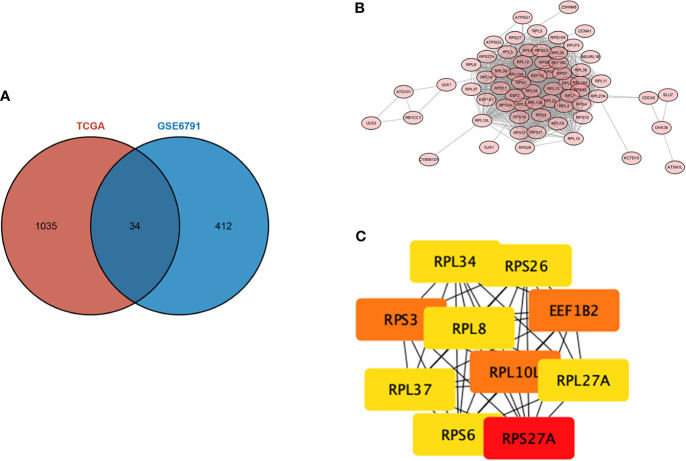
Protein–protein interaction (PPI) network of the differentially expressed genes (DEGs). **(A)** intersecting genes in TCGA and GSE6791. **(B)** PPI constructed using the STRING database. **(C)** Degree ranking of the top 10 potential key genes.

The intersection of the DEGs screened from TCGA and GSE6791 was taken, and a total of 34 overlapping genes were screened, as shown in the Wayne diagram (**Figure 3A**). To analyze the interactions between DEGs, we constructed a PPI using the STRING database and obtained the genes with an integrated score of >0.4 (**Figure 3B**), among which the top 10 pivotal genes in degree ranking were *RPS27A, RPS3, EEF1B2, RPL10L, RPL27A, RPL34, RPS6, RPS26 RPL8*, and *RPL37* (**Figure 3C**).

### *RPS27A* Is a Key Gene

**Figure 4:**
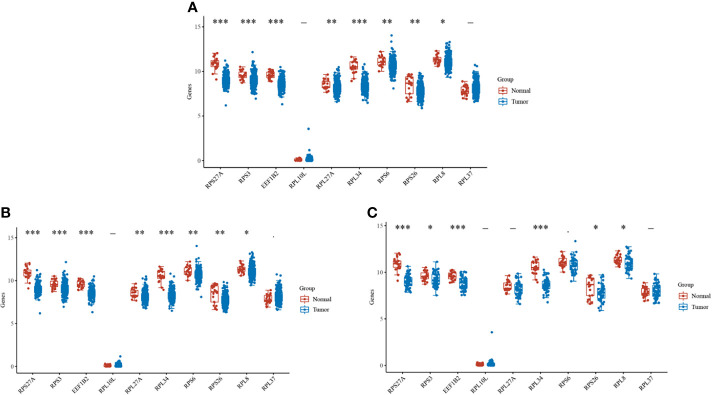
Expressions of the 10 key genes in different cervical cancer types. **(A–C)** Differences in the expressions of the key genes in cervical cancer, cervical phospho-cellular carcinoma, cervical adenocarcinoma, and normal tissues, respectively. *P < 0.05; **P < 0.01; ***P < 0.001.

Cervical cancer was divided into cervical SCC and cervical adenocarcinoma. By observing the difference in the expression levels of key genes in the different types of cervical cancer and normal tissues, we found that most genes, such as *RPS27A, RPS3*, and *EEF1B2*, were significantly expressed in cervical cancer (**Figure 4**). Subsequently, Kaplan–Meier analysis was performed on 10 pivotal genes, and only *RPS27A* was significantly associated with the prognosis of cervical cancer (**Table 1**). Therefore, *RPS27A* was designated as a pivotal gene.

**Table 1:** Summary of the Kaplan–Meier curve data for the 10 key genes.

Genes	*p*-value	HR	Low 95%CI	High 95%CI
*RPS27A*	0.015882274	1.791330669	1.115359057	2.876979882
*RPS3*	0.158973292	1.397120997	0.877287386	2.224980218
*EEF1B2*	0.26483894	1.301492142	0.818991291	2.068253733
*RPL10L*	0.416411021	0.824521873	0.517726551	1.313118512
*RPL27A*	0.6639216	1.10829083	0.696995342	1.76229092
*RPL34*	0.377287049	1.232092918	0.77521818	1.958226726
*RPS6*	0.720160167	1.088208158	0.685267336	1.728080319
*RPS26*	0.508204272	1.169684167	0.735239992	1.860836006
*RPL8*	0.099889699	1.477767994	0.928054852	2.353091781
*RPL37*	0.2883298	1.285697073	0.808499074	2.044550223

### Diagnostic and Prognostic Value of *RPS27A* Expression Level for HPV16

**Figure 5:**
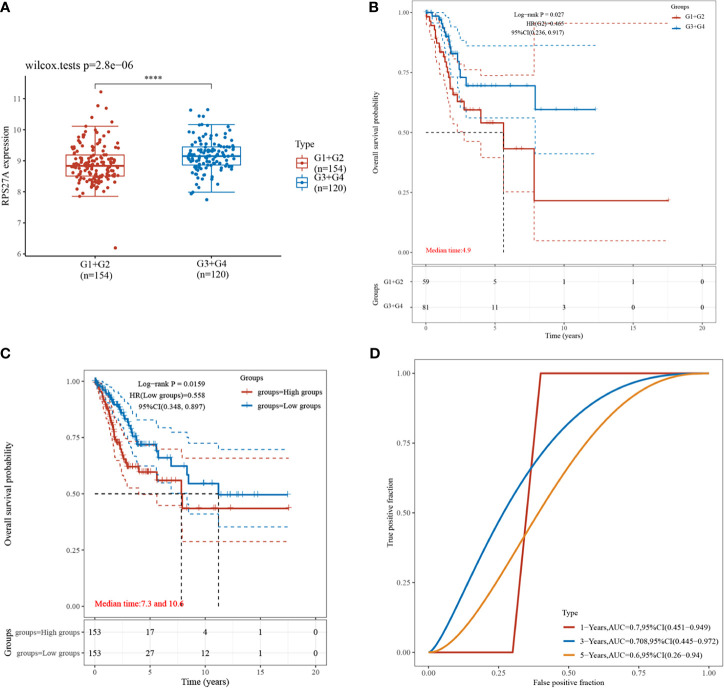
Clinical expression comparison and prognostic analysis of *RPS27A*. **(A)** Comparison of the expressions in different clinical stages, ****P < 0.0001. **(B)** Prognostic impact of a high *RPS27A* expression in clinical analysis. **(C)** Kaplan–Meier curve distribution of high and low *RPS27A* expressions. **(D)** Receiver operating characteristic (ROC) curve of the different survival times of *RPS27A*.

Validation of *RPS27A* revealed significant differences in its expression during the different clinical stages of cervical cancer (**Figure 5A**), and a high *RPS27A* expression was associated with poorer prognosis in patients with advanced cervical cancer (*p* = 0.0023) (**Figure 5B**). In addition, survival analysis showed that cervical cancer patients with a high *RPS27A* expression had worse prognosis (**Figure 5C**), and *RPS27A* expression was associated with poorer prognosis in patients with HPV16-positive cervical cancer at 1 year (AUC = 0.7, 95%CI = 0.451–0.949), 3 years (AUC = 0.708, 95%CI = 0.445–0.972), and 5 years (AUC = 0.6, 95%CI = 0.26–0.94), which were predictive of prognostic survival (**Figure 5D**).

### Biological Characteristics of *RPS27A*

**Figure 6:**
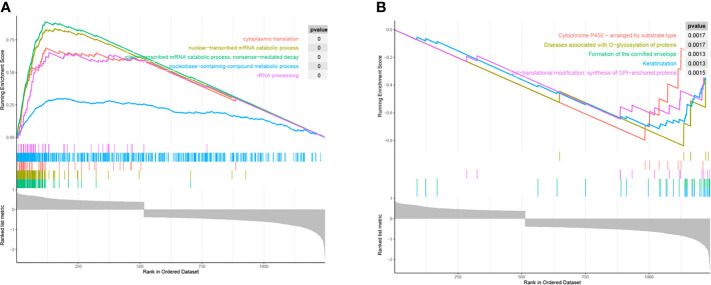
Functional mining of *RPS27A*. **(A)** Gene Ontology (GO) terms of *RPS27A*. **(B)** Biological pathway of *RPS27A* in the Reactome gene set.

The results of GSEA showed that *RPS27A* was mainly enriched in GO functions such as cytoplasmic translation, nuclear–transcriptional mRNA catabolic processes, and ribosomal RNA (rRNA) processing (**Figure 6**). It was also associated with cytochrome P450 (CYP450) arrangement by substrate type, keratinized envelope formation, post-translational modifications: GPI-anchored protein synthesis, and other biological pathways.

### *RPS27A* Is Significantly Expressed in Most Tumours

**Figure 7:**
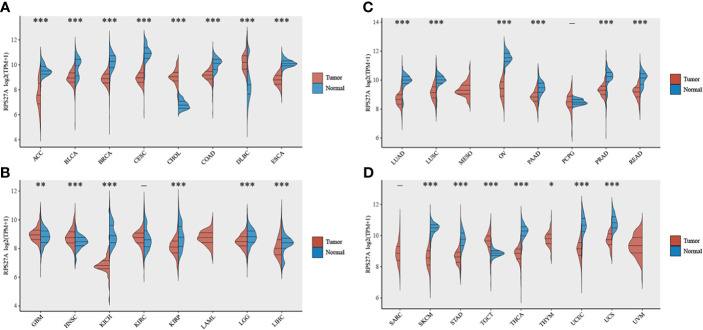
Expression levels of RPS27A in different tumor tissues. **(A–D)** Differences in RPS27A expressions in 33 different tumor tissues and normal tissues in The Cancer Genome Atlas (TCGA). *p < 0.05, **p < 0.01, ***p < 0.001.

As shown in **Figure 7**, *RPS27A* expression was significantly associated with the majority of tumors, such as adrenocortical carcinoma (ACC; *p* < 0.001), bladder urothelial carcinoma (BLCA; *p* < 0.001), breast invasive carcinoma (BRCA; *p* < 0.001), and CESC (*p* < 0.001).

## Discussion

HPV infection is a major cause of cervical carcinogenesis, and repeated infections with the same species of HPV genotypes have a synergistic effect in inducing cervical carcinogenesis (22). HPV encodes two oncoproteins, E6 and E7, and their sustained expression promotes cervical carcinogenesis (23). In particular, HPV16 infection shows a high prevalence in cervical cancer cases, with most infections manifesting without symptoms (24). This study aimed to discover the genes associated with HPV16 cervical cancer and to provide biomarkers for the diagnosis, treatment, and prognosis of cervical cancer.

Ribosomal protein S27A (*RPS27A*), the only key gene screened in this study associated with prognostic survival in cervical cancer patients, belongs to the ribosomal protein S27AE family. It is a component of the ribosomal 40S subunit and is involved in the encoding of the ubiquitin carboxyl terminus (25). *RPS27A* is an RNA-binding protein that performs extra-ribosomal functions, including ribosome biosynthesis and post-translational modification processes (26). It has been documented that *RPS27A* is a direct transcriptional target of p53. It is overexpressed in DNA damage and in kidney, breast, and colon cancers (27) and has roles in promoting proliferation, regulating cell cycle progression, and inhibiting apoptosis (28). In addition, *RPS27A* is involved in the progression of several diseases or cancers: it may be a potential target for Epstein–Barr virus (EBV)-induced LMP1-positive cancer cells (29), its upregulated expression promotes colorectal cancer cell growth and inhibits apoptosis (30), it is involved in the pathogenesis of diabetic pancreatic ductal adenocarcinoma (PDAC) (25), and it is also one of the pathway links that promote the proliferation of HPV immortalized cervical epithelial cells (H8), which can promote cervical carcinogenesis (26). In the present study, a high expression of *RPS27A* could lead to poor prognosis in patients with advanced cervical cancer, serve as a prognostic survival predictor in patients with HPV16-positive cervical cancer, and act as an oncogene in the development of HPV16 cervical cancer.

GSEA showed that *RPS27A* is also associated with GO functions such as cytoplasmic translation, nuclear–transcriptional mRNA catabolic processes, and rRNA processing, all of which are associated with ribosomes. Ribosome biogenesis is a tightly regulated cellular process that begins in the nucleolus and is subsequently processed into rRNA (31). When disrupted during ribosome biogenesis, it can differentially promote cell cycle arrest, senescence, or apoptosis (32). In recent years, the role of ribosomes in carcinogenesis has been extensively validated, linking their involvement in cell cycle regulation and p53 activation to cancer progression (33). For example, *RPS19*, *RPS21*, and *RPS24* can be used as biomarkers for prostate cancer (34), and ribosome dysfunction is associated with the pathogenesis of nasopharyngeal carcinoma (35) and can promote breast cancer metastasis (36).

In addition, CYP450 is also enriched for significant biological pathways. It is a large, intact membrane-conserved superfamily that includes 57 coding genes, mainly found in hepatocytes and enterocytes, involved in the metabolism of cholesterol, oestrogen, vitamin D, and arachidonic acid (37). Li et al. showed that HPV integrated genes strongly prefer the CP450 pathway (38), which is consistent with the results of the present study. Moreover, CP450 is one of the factors that predispose patients to cervical cancer. Studies by several scholars have shown that polymorphic variants in the CP450 family gene *CYP1A1* can increase the risk of cervical cancer, especially in Asians (39, 40). In addition, CP450-related genes also play important roles in other cancers: *CYP4Z1* is involved in regulating breast cancer progression (41), the *CYP17* inhibitor prevents the growth of prostate cancer cells (42), *CYP24A1*, a proto-oncogene in human lung cancer, has anti-differentiation and anti-proliferative effects in human lung cancer cell lines (43), and *CYP1B1* causes apoptosis in neural cancer cells by inducing melatonin (44). This shows that CP450 is important in cancer cell differentiation, proliferation, and apoptosis. The above analysis indicates that the functions between *RPS27A* and CP450 have overlapping parts, suggesting that *RPS27A* might have a synergistic effect with CP450.

Previously, comprehensive bioinformatics analysis methods, such as functional enrichment analysis, PPI network construction, and survival analysis, were used to screen DEGs using the GEO database or TCGA database to identify key genes for cancer progression. For example, Sun et al. (45) and Liu et al. (46) identified biomarkers associated with gastric cancer (GC) progression using this method. In this study, we identified the key gene for HPV16 cervical cancer as *RPS27A*, developed a survival prediction model to confirm its predictive ability, and performed a GSEA to investigate its functional pathway. In conclusion, this study identified *RPS27A* as a key gene for HPV type 16 cervical cancer using a comprehensive bioinformatics analysis approach and that it has an accurate predictive ability for patients’ prognostic survival. Although systematic bias may have arisen due to the large variation in sample size, our findings still provide therapeutic targets with clinical significance for HPV16-associated cervical cancer.

## Data Availability Statement

The original contributions presented in the study are included in the article/supplementary material. Further inquiries can be directed to the corresponding author.

## Author Contributions

All authors listed have made a substantial, direct, and intellectual contribution to the work and approved it for publication.

## Funding

This work was supported by Ningbo Medical and Health Care Brand Discipline (PPXK2018-06).

## Conflict of Interest

The authors declare that the research was conducted in the absence of any commercial or financial relationships that could be construed as a potential conflict of interest.

## Publisher’s Note

All claims expressed in this article are solely those of the authors and do not necessarily represent those of their affiliated organizations, or those of the publisher, the editors and the reviewers. Any product that may be evaluated in this article, or claim that may be made by its manufacturer, is not guaranteed or endorsed by the publisher.
